# Superhydrophilic Polyurethane/Polydopamine Nanofibrous Materials Enhancing Cell Adhesion for Application in Tissue Engineering

**DOI:** 10.3390/ijms21186798

**Published:** 2020-09-16

**Authors:** Kamil Kopeć, Michał Wojasiński, Tomasz Ciach

**Affiliations:** 1 Faculty of Chemical and Process Engineering, Biomedical Engineering Laboratory, Warsaw University of Technology, Waryńskiego 1, 00-645 Warsaw, Poland; michal.wojasinski@pw.edu.pl (M.W.); tomasz.ciach@pw.edu.pl (T.C.); 2 Centre for Advanced Materials and Technologies CEZAMAT, Warsaw University of Technology, Poleczki 19, 02-822 Warsaw, Poland

**Keywords:** polydopamine, nanofibers, polyurethane, hydrophilization, tensile properties, fibroblasts, cell adhesion, tissue engineering, scaffolds

## Abstract

The use of nanofibrous materials in the field of tissue engineering requires a fast, efficient, scalable production method and excellent wettability of the obtained materials, leading to enhanced cell adhesion. We proposed the production method of superhydrophilic nanofibrous materials in a two-step process. The process is designed to increase the wettability of resulting scaffolds and to enhance the rate of fibroblast cell adhesion. Polyurethane (PU) nanofibrous material was produced in the solution blow spinning process. Then the PU fibers surface was modified by dopamine polymerization in water solution. Two variants of the modification were examined: dopamine polymerization under atmospheric oxygen (V-I) and using sodium periodate as an oxidative agent (V-II). Hydrophobic PU materials after the treatment became highly hydrophilic, regardless of the modification variant. This effect originates from polydopamine (PDA) coating properties and nanoscale surface structures. The modification improved the mechanical properties of the materials. Materials obtained in the V-II process exhibit superior properties over those from the V-I, and require shorter modification time (less than 30 min). Modifications significantly improved fibroblasts adhesion. The cells spread after 2 h on both PDA-modified PU nanofibrous materials, which was not observed for unmodified PU. Proposed technology could be beneficial in applications like scaffolds for tissue engineering.

## 1. Introduction

Unique properties of materials composed from submicron fibers, or so-called nanofibers, increased the scientific output in the subject, but we still struggle to transfer such materials into practical applications [1]. The properties of the nano- and submicron scale fibrous materials, like high specific surface area, high porosity, better mechanical properties compared to bulk materials, and overall structural characteristics are associated with their size, and they create an opportunity to improve already existing products, or introduce new products with superior properties [2,3,4], from smart materials (self-healing, self-cleaning materials), through materials for environmental protection (air purification, wastewater treatment), energy and catalysis applications to biomedical applications (wound dressing, tissue engineering) [5,6,7,8,9,10,11,12,13]. The range of applications of submicron fibrous materials depends not only on their properties, but also on the efficient and reliable methods of production. In the last decades, processes like drawing, self-assembly, synthesis on the template, phase separation, and especially electrospinning allowed for the introduction of fibrous materials in various laboratories [14]. To accelerate the industrial adaptation of submicron fibrous materials, scientists introduced the solution blow spinning (SBS), centrifugal spinning, and draw spinning into the field of fibers production [15]. Although the solution blow spinning process is based on the electrospinning (and meltblowing—the most common industrial way of synthetic fibers production [16]), it is from two to one hundred times more efficient than electrospinning [17]. The main difference between electrospinning and solution blow spinning processes lies in the source of the driving force for fibers stretching. In solution blow spinning, the polymer solution nozzle is inner in a concentric nozzles system. Through the outer nozzle of the system flows the compressed gas that, during rapid decompression at the outlet of the nozzles system, increases the velocity and drags—due to viscous interactions—the polymer solution droplet. Dragging the droplet causes its deformation into the cone. From the cone’s apex, the polymer solution jet erupts and is stretched into the fiber. Since solution blow spinning generates a high driving force, the polymer solution feed rate can exceed the one typical for electrospinning by one or two orders of magnitude [17]. What is more, the driving force of solution blow spinning comes from the stream of compressed gas. In general, production facilities are equipped with compressed gas systems more often than in high-voltage systems, necessary for electrospinning. All of that makes the solution blow spinning the most efficient and scalable way of nano- and submicron fibers production in nozzle-based systems [15].

Since the production of nano- and submicrometric fibrous materials reaches the output scale large enough to fulfill commercial needs, such materials find applications in various fields, as mentioned above. In the case of self-cleaning materials, submicron fibrous mats for water purification, and nanofibrous scaffolds for tissue engineering regardless of the structural and mechanical properties, fibers need to be hydrophilic or superhydrophilic. In general, either electrospun or solution blow spun fibers consist of synthetic polymers, mostly hydrophobic. This renders the surface modification processes necessary for the successful use of synthetic polymeric nano- and submicrometric in the mentioned applications. Scientific literature provides numerous methods for changing and controlling surface properties of nanofibrous materials, especially in terms of controlled hydrophilicity and hydrophobicity [18,19]. Such methods include modifications like plasma treatment [20,21], wet chemical method [22], surface graft polymerization [23], co-spinning, target molecule loading [24,25], and polydopamine (PDA) bioinspired coating [26]. Among those methods, PDA coating exhibits high effectiveness and repeatability at a relatively low cost.

PDA attracts interest as a multifunctional coating for many applications, since its first report in 2007 [27]. PDA is a biocompatible, biomimetic polymer that can create a stable coating on virtually any material, hydrophilize its surface and be a base for the covalent attachment of many chemical compounds via thiol or amine groups in the simple water-based process [28,29,30]. PDA can be synthesized from dopamine by three methods: solution oxidation, enzymatic oxidation, or electropolymerization. Solution oxidation in an alkaline environment using atmospheric oxygen as an oxidizing agent is the most commonly used [28]. In most cases, the synthesis is carried out for 24 h, which allows obtaining a stable coating with a thickness of about 50 nm [27,31]. However, dopamine polymerization can be much faster and can be possible also in a neutral or acidic environment when oxidants other than atmospheric oxygen (e.g., ammonium persulfate, copper sulfate, hydrogen peroxide, sodium periodate) are added. Oxidant-induced PDA synthesis also allows for obtaining thicker coatings [31,32,33,34]. What is more, the chemical structure of PDA films differs depending on the process conditions such as buffer composition and concentration, pH, temperature, addition, and the concentration of oxidizing agents or surfactants [27,34,35]. The structure of PDA is very complex and consists of a mixture of various oligomers that are products of dopamine oxidation, including indole units with different degrees of oxidation and open-chain dopamine units [36]. Many functional groups are present in PDA films, including planar indole units, amino groups, carboxylic acid groups, catechol or quinone functions, indolic/catecholic π-systems [37]. The presence and concentration of specific functional groups depend on the process conditions mentioned above. These differences in the chemical composition of PDA synthesized in different ways may affect the properties of the obtained coatings.

It has been proven several times that PDA coating promotes the adhesion and proliferation of many types of mammalian cells, e.g., myoblasts, hMSCs, HUVECs, chondrocytes, HT1080 cells, on different materials, e.g., polyurethane (PU), polydimethylsiloxane (PDMS), polycaprolactone (PCL), poly(lactic-*co*-glycolic acid) (PLGA), poly(L-lactic acid) (PLLA), glass, stainless steel and titanium [38]. Less attention was given to the analysis of the process of cell adhesion to the PDA-modified materials, with a short contact time between the cells and material surface. Wang et al. investigated the adhesion of HUVECs to PDA-coated PCL films. They observed that PDA coating promotes the attachment and spreading of cells after 0.5 h of contact with the material, while on uncoated PCL films, this phenomenon was not observed after 4 h of the culture [39]. Ku et al. showed that HT1080 cells can attach and spread on PDA-coated PDMS after 2 h of the culture. The cells grew selectively only in areas of the material coated with PDA [40]. There is still a gap in the knowledge regarding the adhesion and morphological changes of mammalian cells on PDA-coated fibrous materials in short cultivation times (less than 2 h).

Air oxidizing PDA coatings were used for the hydrophilization of different electrospun nano- or microfibrous materials made of poly(lactic acid)/cellulose composite [41], polycaprolactone [42], poly(lactide-*co*-glycolide) [43], polyacrylonitrile, polysulfone [44] and polyurethane [45]. Most of them were prepared as a scaffold for tissue engineering. Coating the surface with PDA resulted in fibers surface hydrophilization of all materials reported. However, there is still a gap in studies on the modification of the fibrous materials by PDA coatings synthesis in the fast process with the addition of oxidants. The modification of fibers produced by the solution blow spinning method and stability of the hydrophilic properties of PDA-modified fibrous materials has also not been tested.

This paper describes the development of an efficient and reliable process of production of superhydrophilic nanofibrous materials in two steps, but under 3 h. As a result, we aimed at significantly increasing the cell’s adhesion rate to the surface of modified polyurethane nanofibers. We used the solution blow spinning method to produce fibrous structures from polyurethane. Then, we examined two variants of the fibers modification process and evaluated their impact on the morphological and mechanical properties, and mostly on the hydrophilicity of the fibrous material. Finally, we examined the effect of modifications on the L929 cells adhesion rate in the first hours of contact with the PDA-modified materials.

## 2. Results and Discussion

**Figure 1:**
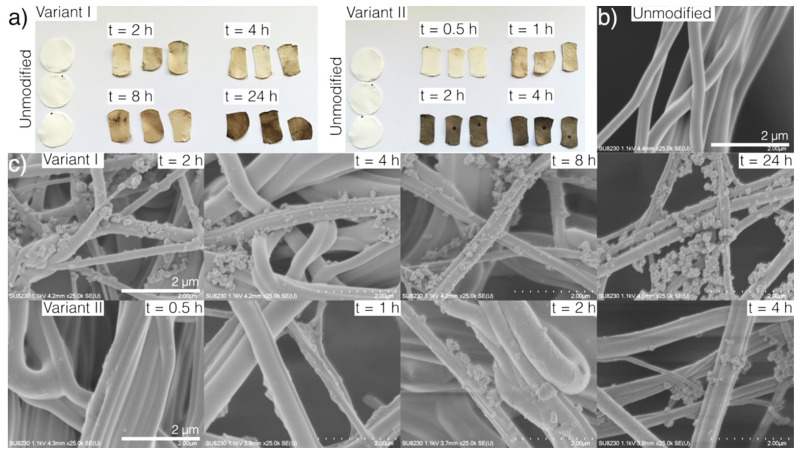
(**a**) pictures of unmodified and modified polyurethane (PU) nanofibrous materials, scanning electron microscopy (SEM) images of (**b**) unmodified, and (**c**) modified PU nanofibrous materials.

Modification of the PU nanofibrous material by forming PDA coating results in a color change of the material from white through brown to black, as shown in Figure 1a. Color intensity increases with process duration for both tested variants.

As we can observe in SEM images (Figure 1c), a uniform layer of PDA, as well as some PDA nanoparticles, are deposited on the surface of the pristine fibers (Figure 1b) as a result of the modification process. The formation of PDA nanoparticles during the dopamine hydrochloride oxidative polymerization is a known phenomenon [46]. The number of deposited nanoparticles increases with the modification time, probably due to the diffusional transport of nanoparticles to the surface of the nanofibers. We observed that PDA nanoparticles already adhered to the fibers are consequently coated with the PDA layer that is forming during the process. These nanoparticle-based dendritic structures increase the specific surface area of the nanofibrous material, which may be advantageous for some applications, e.g., bioactive compounds immobilization or cell proliferation.

After a long process time in variant I of modification (V-I, described in Section 3.2), we can observe a segmented structure of the PDA coating on single fibers, which is not found in variant II (V-II, also described in Section 3.2) (Figure 1c). This phenomenon does not depend on the thickness of the coating, and may be the result of differences in the chemical composition of the PDA synthesized by two tested methods, and may affect the different physical properties of the obtained nanofibers, as previously described [34].

**Figure 2:**
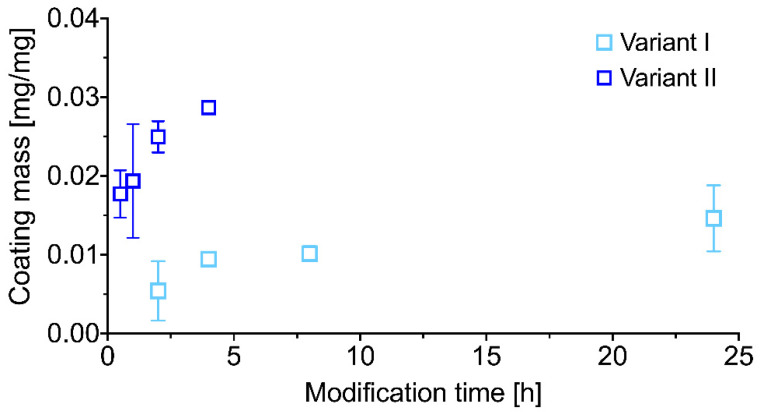
Coating mass increment versus modification time for PU/polydopamine (PDA) modified nanofibrous materials.

**Figure 3:**
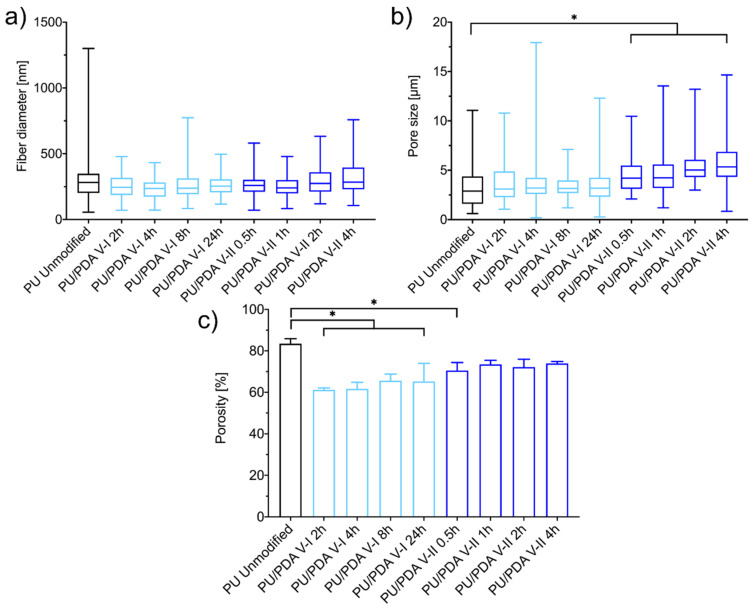
(**a**) size distributions, (**b**) pore size distributions, and (**c**) porosity of unmodified and modified PU nanofibrous materials. The asterisk (*) denotes a significant (*p* < 0.01) difference among the mean value of the characteristics of nanofibrous materials.

The mass of the coating synthesized on the surface of PU nanofibers increases with time of the modification process, regardless of the modification variant, as expected. What is evident, the mass increment in variant II is much steeper comparing to variant I. What is more, the coating reaches a similar mass in variant II after only 0.5 h as in variant I after 24 h (Figure 2). However, the described increase in scaffolds mass did not affect the structural and morphological properties of PU nanofibrous materials (Table 1, Figure 3).

Pristine solution blow spun PU fibrous materials exhibit the mean fiber diameter at about 300 nm, with a mean pore size of about 3.5 μm and porosity about 85% (Table 1). Due to the PDA modification process, depending on the modification variant, those properties change, except for the mean fiber diameter (Figure 3a). The most significant change among the investigated structural properties of the PU fibrous material can be observed in porosity (Figure 3c). V-I results in about 20 percentage points reduction of the porosity, while V-II of the modification processes reduces the porosity twice as much. Furthermore, the mean pore size of the PU fibrous material increases significantly after modification using the V-II, especially after two and four hours (Table 1, Figure 3b). Both effects, the increase of pore size and decrease of porosity, come from the contraction of the PU materials during the drying step after the modification process (reduction of materials thickness, Table 1). Dried material exhibits more fibers stuck together, resulting in lower thickness and lower total volume of the material and more of the free space between fibers. Only ten percentage points reduction of the mean porosity, with the increase of the mean pore size of the PU fibrous materials, can be considered as favorable in many applications of such materials (e.g., filtration), especially in the application in tissue engineering as scaffolds for guided cell growth [12,47].

**Figure 4:**
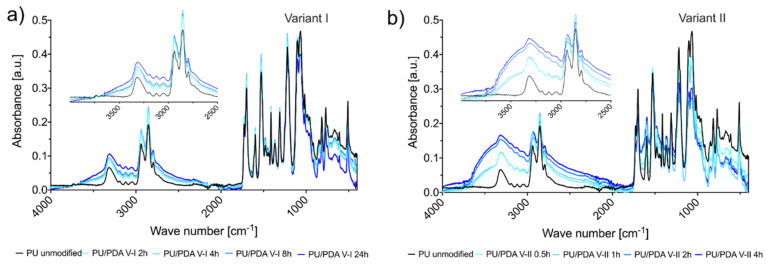
Fourier-transform infrared spectroscopy with attenuated total reflection (FTIR-ATR) spectra of nanofibrous materials in function of modification time in (**a**) variant I, and (**b**) variant II. Inserts enlarge the wave number range from 2500 to 4000 cm^−1^.

Figure 4 shows that the FTIR-ATR spectra of PDA-modified PU nanofibrous materials contain a broad band from 3400 cm^−1^ to 3700 cm^−1^ that is ascribed to the N-H and catechol -OH groups, which were brought by PDA, and made the material hydrophilic [48]. This band is not observed in the spectra of unmodified material, which indicates that PDA successfully coated the materials. The absorption in the 3400–3700 cm^−1^ region increases with the modification time for both tested variants, which confirms that a longer modification allows obtaining thicker coatings. The higher absorbance in this region for modification variant II means that the process using sodium periodate as an oxidizing agent allows one to obtain thicker coatings in a shorter time compared to polymerization using atmospheric oxygen (variant I). Such a result corresponds with the observation of higher coating mass in variant II (Figure 2).

**Figure 5:**
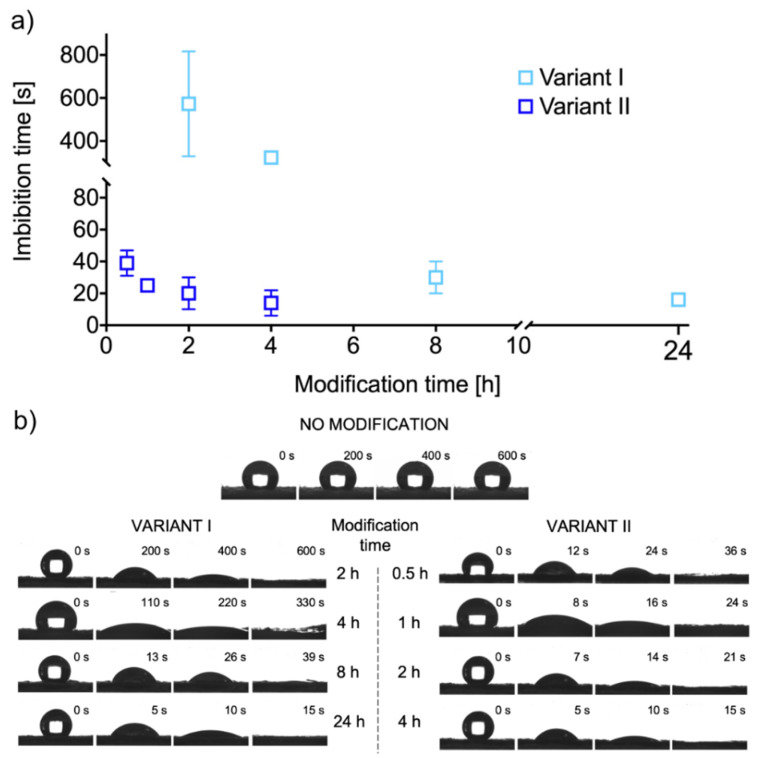
(**a**) Water droplet imbibition time as a function of PU nanofibrous materials modification time for both modification variants, (**b**) images of water droplet shape changes during the imbibition process on the surface of unmodified and modified PU nanofibrous materials.

Figure 5 shows the effect of PDA coating on the PU nanofibrous material hydrophilization. The unmodified material has strongly hydrophobic properties, with a water contact angle of 131 ± 3° stable over time, while the modified material has superhydrophilic properties (Figure 5b). In the modified material, both external and internal fibers are highly wettable, which results in the imbibition of a water droplet from the surface of the fibrous material. We used the measured water droplet imbibition time to differentiate the hydrophilization efficiency of a polyurethane nanofibrous material depending on the method and modification time.

We applied two methods for the synthesis of PDA coating on the PU nanofibers, using atmospheric oxygen (V-I) and sodium periodate (V-II) as oxidizing agents (details in Section 3.2). In V-II, the process was conducted at pH 5.5, in which the polymerization of dopamine hydrochloride does not occur spontaneously with the presence of atmospheric oxygen only; a phenomenon exists at pH 8.5 in V-I [32]. Wettability, measured by the time of water droplet imbibition from PU fibrous material surface, is a function of modification time for both tested variants (Figure 5a). After 24 h of the modification process in V-I, we obtained the water droplet imbibition time similar to that after 4 h of modification in V-II (16 ± 4 s and 14 ± 8 s, respectively). These results confirm previous reports that the use of sodium periodate significantly increases the rate of dopamine polymerization [34]. The tested material was superhydrophilic, even for a short modification time (0.5 h, V-II, see Video S3), compared to the less hydrophilic material obtained for the shortest tested modification time in V-I (2 h, V-I, see Video S2). High values of standard deviations may result from irregular PDA coating and nanoparticle-based dendritic structures on the surface of fibers or non-uniform packing of fibers in the structure of the entire nanofibrous material.

As we stated previously, the other authors used PDA coatings for hydrophilization of different fibrous materials. The ability to imbibe a water droplet from the surface has been observed for all of these PDA coated materials, except for the pristine PU nanofibrous scaffolds [28,41,42,43,44,45]. Davoudi et al. prepared PDA-modified PU nanofibrous scaffolds, in which the water contact angle was reduced from 125.2 ± 3.9° to 75.4 ± 3.5° in reference to the unmodified material [45]. They produced a PDA coating on the surface of the nanofibers in the reaction conducted at pH 8.5 for 1 h. Probably, the reaction time was too short for the material to be able to imbibe water inside its structure. In this study, we proved that PU fibrous materials could also be superhydrophilized by surface modification with PDA coating, and the use of sodium periodate as an oxidizing agent can shorten this process to less than one hour. Additionally, the material coated with PDA from polymerization reaction with both oxidizing agents has the ability to imbibe the water droplet from the surface to the volume of the material.

In the introduction, we showed that scientific literature provides information about the different combinations of production and modification (post-processing) strategies for the manufacturing of hydrophilic or superhydrophilic nanofibrous materials. Our approach to the two-step process of production of superhydrophilic nanofibrous materials allows us to prepare the suitable material in even 1 h (excluding the drying step at the end). Huang et al. went for electrospinning of polyacrylonitrile and polysulfone nanofibrous mats over the time of 4 h per mat [44]. Compared to the solution blow spinning process described in this paper, the approach of Huang and co-workers was from 8 to 10 times less efficient in terms of the production time of nanofibrous mat only. However, they used PDA coating (without any oxidizing agents) to further hydrophilize their materials, which resulted in the imbibition of the water droplet in water contact angle measurements. Nevertheless, combining electrospinning and PDA coating in the work of Huang et al. allowed the production of superhydrophilic mats within the time range from about 5 to 22 h (excluding the drying step at the end) [44]. The least efficient step in most approaches to superhydrophilic nanofibrous materials production is the preparation of a nanofibrous mat. In most cases, the electrospinning remains the first method of choice. However, Mercante et al. [21] and Ko et al. [23] showed that, with appropriate post-processing methods, nanofibrous materials for further hydrophilization could be successfully and efficiently produced using solution blow spinning. Nevertheless, Mercante with co-workers [21] decided to use a multi-step modification process that includes 48 h long reduction of graphene oxide coating. In contrast, Ko with the team [23] used not only a multi-step modification process, but in order to bind the pendant oligo(ethylene glycol)-containing polymethacrylate to their fibrous material, they decided to synthesize their polylactic acid. The second approach required five steps in order to prepare the nanofibers for modification and to purify the coating after reaction (final drying excluded) [21,23].

**Figure 6:**
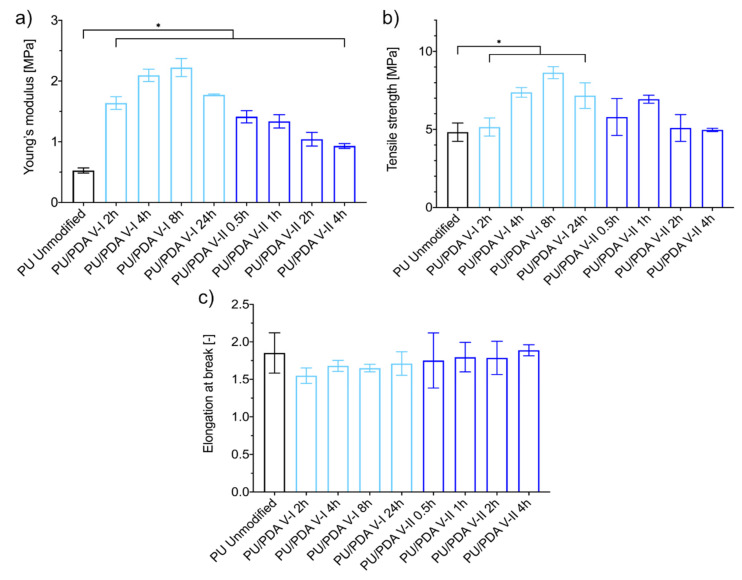
Mechanical properties of unmodified and modified PU nanofibrous materials: (**a**) Young’s modulus, (**b**) tensile strength, and (**c**) elongation at break. The asterisk (*) denotes a significant (*p* < 0.01) difference among the mean value of the mechanical properties of nanofibrous materials.

**Figure 7:**
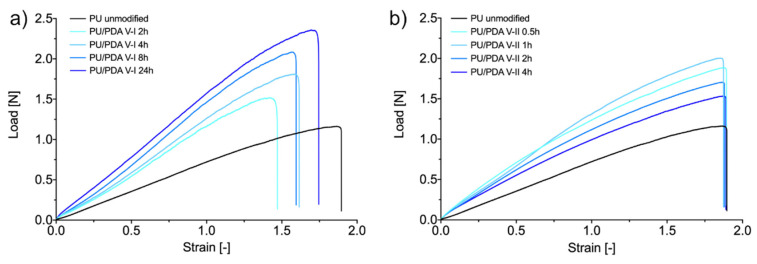
Representative load/strain curves of unmodified and modified PU nanofibrous materials: (**a**) variant I, (**b**) variant II.

Unmodified PU nanofibrous mats exhibit Young’s modulus of 0.527 ± 0.043 MPa, the tensile strength of 4.820 ± 0.588 MPa, and elongation at break of 185 ± 27%. After treatment, according to protocols of variant I and variant II for the increase of hydrophilicity of PU nanofibers, most of the materials’ tensile properties changed. Both variants led to a significant increase in materials stiffness (Young’s modulus). Modification according to V-I increases the modulus from 3 to 4 times, while the V-II process results in a 2 to 3-fold increase of this property (Figure 6a). In Figure 6a, we can observe the maximum value of the modulus for V-I modification (2.220 ± 0.149 MPa). The tensile strength of the modified materials increases in a similar way as Young’s modulus; however, only the change observed as a result of V-I treatment is significant. Again, modification according to V-I results in the maximum value of material tensile strength (8.640 ± 0.387 MPa) (Figure 6b). This local maximum suggests the presence of the optimal conditions for PU nanofibers modification in V-I, in terms of the increase of the tensile properties of such materials. The occurrence of maxima in V-I in an 8-h process can be caused by the presence of a segmented structure of PDA coating on single fibers in long-term modification. The occurrence of narrow gaps in the PDA coating after 24 h of the process probably results in a significant change in mechanical properties. Even though Young’s modulus and tensile strength of PU nanofibrous materials modified using both presented approaches increase, there is a significant change in elongation at break (Figure 6c). We expected the increase of the two tensile properties of nanofibrous materials, without the parallel effect on elongation at break, since Huang et al. presented similar results, but on different polymers (polyacrylonitrile and polysulfone) [44]. Although the improvement of the mechanical properties of PU/PDA nanofibrous materials could be an advantage in applications like filtration, in terms of tissue engineering, scaffolds should have designed mechanical properties. Hence, the presented results (Figure 7) should be taken into account while designing the scaffold for regeneration of the specific tissue [41].

**Figure 8:**
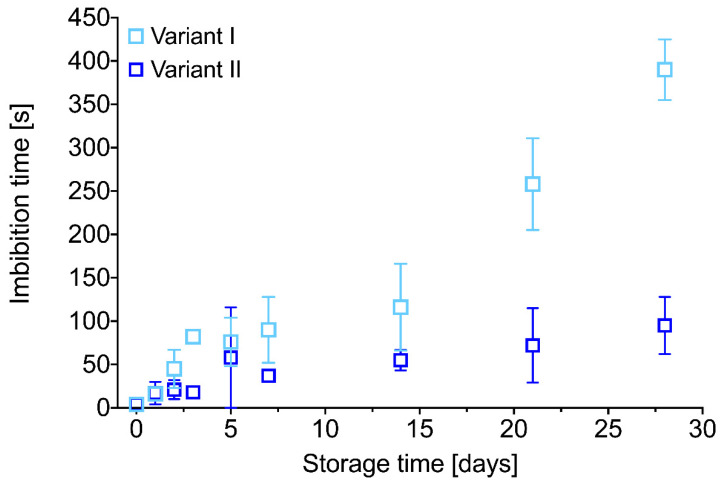
Stability of the hydrophilic property of PDA-modified PU nanofibrous materials (V-I, 8 h of the modification, V-II, 0.5 h of modification).

Figure 8 shows that PDA-modified PU fibrous material has superhydrophilic properties after 28 days of samples’ storage under atmospheric conditions. However, we observed a significant increase in water droplet imbibition time during storage. This may be due to subsequent oxidation reactions in the PDA coating in contact with air or reorganization in the PDA layer, where hydrophobic domains are shifted to the interfacial surface. This phenomenon appears to be like the one observed for the hydrophilization of polymer surfaces by plasma modification [49,50,51]. What is more, variant II of PDA modification on the surface of PU nanofibers results in four times faster water droplet imbibition time (imbibition time of about 100 s) after 28 days of storage of modified materials than on those modified using longer variant I (imbibition time of about 400 s, see Figure 8). This may be due to the different chemical structure of the PDA coatings obtained in each variant, as previously described. Stability of the wetting properties over time remains crucial for the long-term shelf-life of the nanofibrous scaffolds—an expected product for in situ tissue engineering [52].

**Figure 9:**
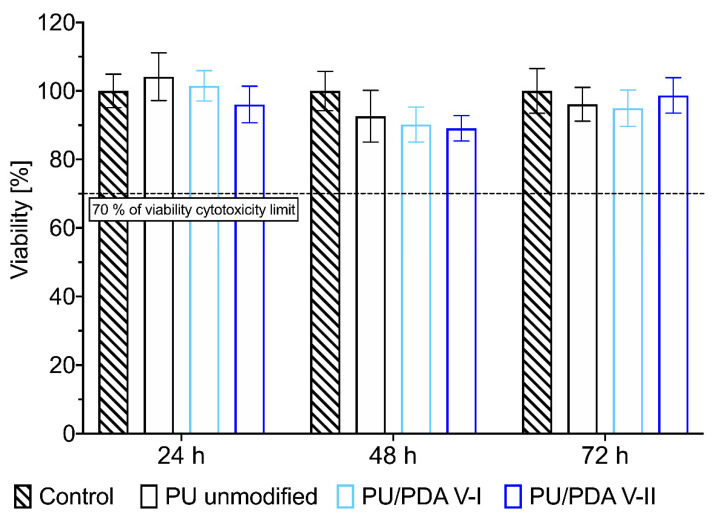
XTT cytotoxicity evaluation of extracts from unmodified PU and PDA-modified (VI—8 h of the modification, VII—0.5 h of modification) nanofibrous materials incubated for 24, 48, and 72 h.

For the cell attachment study, we chose three types of investigated PU nanofibrous materials: unmodified PU, PU modified over 8 h according to variant I (PU/PDA V-I), and PU modified over 0.5 h according to variant II (PU/PDA V-II). However, before the adhesion study, we performed a cytotoxicity evaluation of the chosen materials (details in Section 3.9). Using an indirect extracting approach according to ISO 10993-5:2009, a cytotoxicity test with XTT results in non-cytotoxic properties in vitro of all tested samples (Figure 9). Results over the threshold of 70% of the cell’s relative viability for 72 h of the incubation suggest that both modified fibrous materials remained stable in the extraction medium (in conditions of 37 °C, 5.0% of CO_2_), confirming the results of the coating stability study reported in the previous paragraph. No cytotoxic agent leached from the materials in the investigation period. If nanoparticles present at the surface of modified materials (see Figure 1c) detach from nanofibers, they exhibit no cytotoxic properties in in vitro test when XTT assay is used. What is more, results of the cytotoxicity assay show that there is no SDS (from washing step of scaffold modification, see description in Section 3.2) present within the volume of the PU/PDA modified nanofibrous scaffolds.

**Figure 10:**
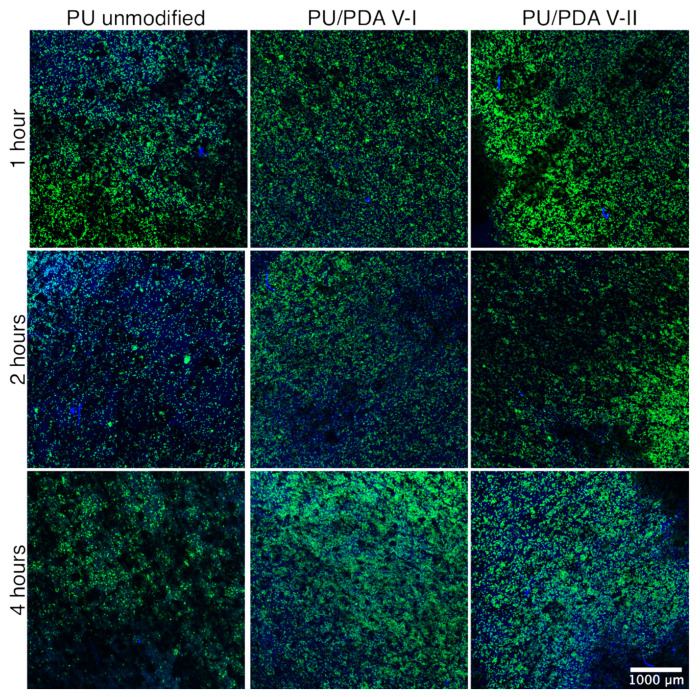
Confocal laser scanning microscope wide-view images of L929 cells attachment on the surface of PU unmodified PU and PDA-modified (VI—8 h of the modification, VII—0.5 h of modification) nanofibrous materials after 1, 2, and 4 h of cells incubation.

Confocal laser scanning microscopy (CLSM) revealed that, regardless of the nanofiber’s modification, L929 cells are present on the surface of the investigated PU and PU/PDA materials. Mosaic images showing about 20 mm^2^ of the surface of the material after 1, 2, and 4 h of L929 cell culture reveal close to total coverage of the materials with living cells (Figure 10). This observation suggests that high surface-to-volume ratio and like extracellular matrix morphology of nanofibrous materials are crucial for proper cell immobilization of the surface of the scaffold. However, increased wettability improves the rate of cell adhesion and increases the area covered by cells at the surface of both modified PU/PDA nanofibrous materials, especially after 4 h of cell culture (Figure 10).

**Figure 11:**
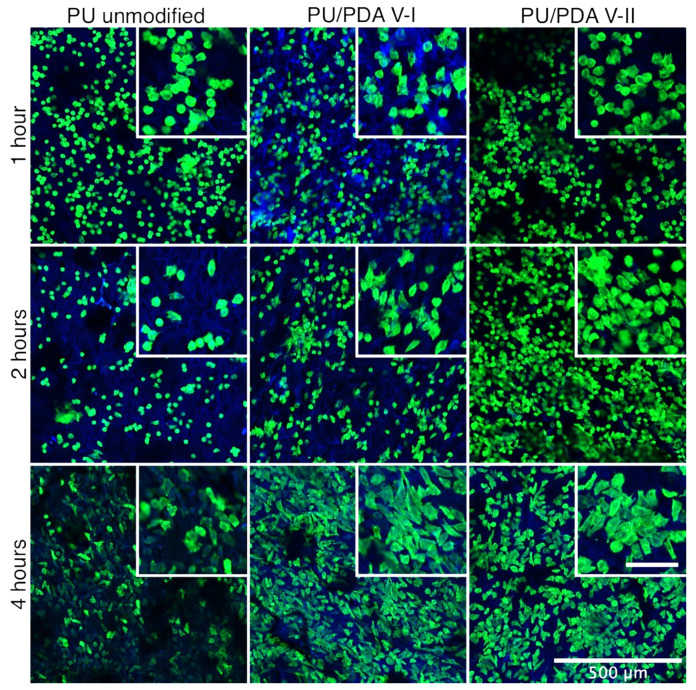
Confocal laser scanning microscope close-up images of L929 cells attachment on the surface of PU unmodified PU and PDA-modified (VI—8 h of the modification, VII—0.5 h of modification) nanofibrous materials, after 1, 2, and 4 h of cells incubation. Insert images enlarge the cells spreading and adaptation to the surface of the modified material. The scale bar in insert image represents 100 μm.

Since cell adhesion molecules are attached to elements of the cell’s cytoskeleton, to investigate L929 cells attachment to the surface of unmodified PU and PDA-modified PU nanofibrous materials, we used higher magnification in CLSM observation (Figure 11). Such close observation of the cells cytoskeleton and spreading of the cells on the surface of materials shows that L929 cells spread similarly on all tested materials after 4 h, which is a typical behavior of fibroblast-like cells [53,54,55]. The first visible change of fibroblasts cultured on PDA-modified PU nanofibers appears after just 1 h of culture (on the PU/PDA V-I). However, a significant difference in cell morphology is observed after 2 h. The cells started spreading on both PDA-modified PU nanofibrous materials, which was not observed for unmodified PU. This effect is more substantial for V-I modification, which may result from the different chemical structure of the PDA coatings obtained in each variant, as previously described. Ku et al. reported a similar observation of human fibrosarcoma (HT1080) cells spreading after 2 to 5 h; however, this was observed using phase-contrast microscopy, without close investigation of the cytoskeleton, on the flat surface of PDA-modified PDMS [40].

## 3. Materials and Methods

### 3.1. Polyurethane Fibers

PU (Elastollan 1185A, BASF, Ludwigshafen, Germany) fibers were produced in the SBS process, described in detail elsewhere [56]. PU solution in pure tetrahydrofuran (≥98.5%, Chempur, Karlsruhe, Germany)—8% *w/w*—was supplied through the inner nozzle of the concentric nozzles system in the SBS apparatus with a flow rate of 30 mL∙h^−1^. At the same time, the airstream was supplied through the outer nozzle of the SBS nozzles system with a pressure of 0.1 MPa. Fibers were produced by shear-drag elongation of the polymer solution by the stream of air on the distance of 30 cm between the nozzles system and the surface of the collector. As a collector, the rotating cylinder was used (cylinder diameter: 10 mm, length: 100 mm, rotational speed: 3000 rpm). Samples were cut from the produced fibrous material into the form of the 16 mm in diameter discs for further modification with PDA and examination.

### 3.2. Polydopamine Coating

PU fibrous discs were rinsed with distilled water and then isopropanol (≥99.7%, Chempur) for 1 min to wet the surface of the fibers and remove air bubbles from the spaces between them. Isopropanol-soaked discs were then immersed in PDA forming solutions. Two variants of fibers’ surface modification process were examined using two different oxidizing agents in dopamine oxidative polymerization: V-I—atmospheric oxygen; V-II—sodium periodate. In V-I, the disks were immersed and stabilized in a 2 mg∙mL^−1^ dopamine hydrochloride (99%, Alfa Aesar, Haverle, MA, USA) solution, in 10 mM Tris-HCl buffer at pH 8.5. In V-II, the disks were immersed in a 2 mg∙mL^−1^ dopamine hydrochloride solution in 10 mM acetate buffer at pH 5.5, with the addition of sodium periodate (BioUltra ≥ 99.5%, Sigma, St. Louis, MO, USA) in a molar ratio of 1:2 to dopamine. Solutions were mixed on a magnetic stirrer (150 rpm) at room temperature for tested periods. After the process, the disks were washed in distilled water with stirring and water exchange every 2 min. The process was carried out until no contamination of unbounded PDA was observed in the water (4 to 6 water exchanges). Then, the disks were washed in 0.1% sodium dodecyl sulfate (pure, 100%, Avantor Performance Materials, Center Valley, PA, USA) solution for 5 min, followed by washing in distilled water for 2 min, and dried at room temperature overnight. The progress of coating synthesis was investigated by weighting the samples, using analytical balance (Excellence XPE205DR/M, Mettler Toledo, Columbus, OH, USA), obtained in every modification time (*n* = 3). Results are presented as the mean value of coating mass per mass of the scaffold ± standard deviation.

### 3.3. Scanning Electron Microscopy

Morphology, fiber size, and pore size of the fibrous materials after production and surface modification with PDA were investigated using SEM. Samples were prepared by cutting off a small portion of the disc and by putting it on the surface of the SEM stub using carbon/aluminum conducting tape. Prior to imaging, samples were coated (K550X Emitech, Quorum Technologies, Laughton, UK) with about 10 nm layer of Au:Pd (60:40 mass ratio). SU 8230 Hitachi FESEM with 1.1 kV accelerating voltage and a working distance of about 4 mm was used. Secondary scattered electrons were detected using the upper detector (SE(U)). Based on the SEM images, the 100 fibers and 100 pores were measured for each sample. Results are presented as the mean value ± standard deviation for fiber size, and the mean value ± standard deviation for pore size.

### 3.4. Porosity

The porosity of the fibrous materials after production and surface modification was determined based on the following relation: P(%) = (1 − d_s_/d_p_) × 100%, where d_s_—sample density, d_p_—polymer density (1.12 g∙cm^−3^). Disc samples were weighed, and sample density was calculated: d_s_ = m/(δ∙A), where m—sample mass (g), δ—sample thickness (cm), A—sample area (cm^2^) [57]. The sample thickness was measured using SEM. Porosity measurement was performed three times, and mean values ± standard deviation are reported.

### 3.5. Fourier Transform Infrared Spectroscopy

The efficiency of PDA coating formation on the surface of fibrous materials was determined by Fourier transform infrared (FTIR) spectroscopy using a Nicolet™ 6700 spectrometer (Thermo Fisher Scientific, Walsham, MA, USA). Spectra were detected in attenuated total reflection (ATR) mode and analyzed with the OMNIC 8.3 software (Thermo Fisher Scientific, Walsham, MA, USA). Spectra were recorded for at least four randomly selected spots for each sample in tested modification time points. One characteristic spectrum for each tested material was selected for presentation.

### 3.6. Wetting Properties

Hydrophilic properties of PDA-modified PU fibers were studied using the sessile drop method (DSA100 goniometer, Krüss GmbH, Hamburg, Germany). Then, 5 μL distilled water droplets were dispensed on the surface of the fibrous material (about 1.6 cm^2^), and the images of droplets were taken every 1 s with the digital camera. The time from the contact of a water droplet with the surface of PDA-modified fibrous material to complete the imbibition of the droplet from the surface to the material structure was defined as a “water droplet imbibition time.” (See measurement Videos S1–S3 in Supplementary Materials) The unmodified fibrous material was used as a control. All measurements were carried out for three independent samples for each tested variant, and water contact angle values and water droplet imbibition time values are presented as mean ± standard deviation.

Time stability of the hydrophilic properties of PDA-modified PU fibrous material was examined by measuring the water droplet imbibition time as a function of material storage time. Selected samples (V-I, 8 h of the modification, V-II, 0.5 h of modification) were stored in closed ventilated Petri dishes under atmospheric conditions at room temperature. After 1, 2, 3, 5, 7, 14, 21, and 28 days of sample storage, the water droplet imbibition time was measured according to the methodology described above.

### 3.7. Tensile Test and Mechanical Properties

Rectangular samples of unmodified fibrous materials and materials modified using both investigated variants of dopamine polymerization coating underwent a uniaxial stretching test according to protocols established based on ASTM standards (designation: D 882-02 and D 638-02a). The experiment was conducted using an Instron 3345 model with a crosshead speed of 10 mm∙min^−1^ at room temperature and humidity. Load-strain curves were measured, as were the maximum load and strain at rupture. It is emphasized that, according to porosity measurements and SEM images, only a fraction of each sample thickness is occupied with fibers (1-P), which implies that the applied load is supported by only such fraction of the sample’s thickness. This effect was accounted for in the data processing for maximum stress calculation. For unmodified PU fibrous materials and each modification variant, three samples were measured, and results are presented as mean values ± standard deviation.

### 3.8. Cell Culture

The mouse fibroblast cell line L929 was purchased from ATCC. Cells were maintained in Dulbecco’s modified Eagle medium (DMEM, Thermo Fisher Scientific, Walsham, MA, USA), and supplemented with 10% fetal bovine serum (FBS, Thermo Fisher Scientific) and antibiotics (100 U mL^−1^ penicillin, 100 mg mL^−1^ streptomycin, Thermo Fisher Scientific). Cells were cultured in 75 cm^2^ cell culture flasks and kept at 37 °C in an incubator with 5% CO_2_. The culture was monitored under the microscope every three days and passaged when it reached near 100% confluency. For cell passage, a trypsin-EDTA solution was used according to standard procedure. Cell concentration was counted on a Bright-Line^TM^ hemacytometer (Cambridge Instruments, Inc., London, UK) using methylene blue staining.

### 3.9. In Vitro Cytotoxicity

A nanofibrous scaffolds extract viability assay was prepared as described in ISO EN 10993-5:2009 [58]. A nanofibrous scaffold made of PU (*n* = 2) and scaffolds modified with PDA according to variant I (8 h—PU/PDA V-I, *n* = 2) and variant II (0.5 h—PU/PDA V-II, *n* = 2) were immobilized by polypropylene insert in 24-well plate and sunk in 1 mL of culture medium for 24, 48 and 72 h for obtaining scaffolds extracts. Supplemented DMEM was used as negative cytotoxicity control (*n* = 2), while a 1.0% solution of Tween 80 in DMEM served as a positive control. L929 cell line was cultured in 96-well plates for 24 h in the concentration of 10^5^ cells mL^−1^ and 100 μL of culture medium for each well. After this time, the culture medium was replaced by extracts and both control samples. After 24 h the extracts and control samples were removed from each well and 100 μL of DMEM, without phenol red and supplementation, and 50 μL of XTT solution with coupling reagent (Cell Proliferation Kit II (XTT), Sigma Aldrich) was added to each culture well and incubated for 4 h. After the incubation with XTT; 100 μL from each well was transferred to a new 96-well plate, and the absorbance at 475 nm was measured in a plate spectrophotometer (Epoch, BioTek, Vinusky, VT, USA). The relative cell viability was defined as the ratio of the absorbance from the sample to the mean absorbance measured for negative control and presented as a mean value ± standard deviation.

### 3.10. Cell Adhesion

For the adhesion study, L929 cells were seeded at a concentration of 10^5^ cells mL^−1^ per scaffold in 1 mL of cells suspension. Prior to cells seeding, scaffolds were immobilized in the well of 24-well plate using polypropylene insert. The experiment was conducted in the same cell culture conditions as described previously for 1, 2, and 4 h. Prior to imaging using CLSM (LSM 880, Zeiss, Sheung kehen, Germany), cells attached to the surface of the scaffolds were rinsed vigorously twice with phosphate-buffered saline (PBS) and prepared using the following protocol. L929 cells on the surface of the scaffolds were fixed with 4% paraformaldehyde (Sigma-Aldrich) solution in PBS and stained using an Alexa Fluor^TM^ 488 Phalloidin (green staining of filamentous actin to visualize cytoskeleton, Invitrogen) and DAPI (blue staining of a nucleus, Invitrogen), according to the protocol provided by the manufacturer. As prepared, samples were analyzed using CLSM.

### 3.11. Statistical Analysis

The normality of distributions of fiber size, pore size, porosity, and mechanical properties results was tested using the Shapiro–Wilk test (*p* < 0.05). The difference among the mean fiber sizes and mean pore sizes of analyzed materials was tested in the Kruskal–Wallis test (*p* < 0.01), with post hoc nonparametric Dunn’s test for multiple comparisons. The difference among the mean porosity and mean values of the mechanical properties of analyzed materials was tested in one-way ANOVA (*p* < 0.01) with post hoc Tukey’s test for multiple comparisons.

## 4. Conclusions

We present the two-step process for the efficient production of superhydrophilic nanofibrous materials from polyurethane, having an increased cell adhesion rate to their surface and improved mechanical properties. Application of the solution blow spinning process in the first step of the presented approach results with at least ten times faster production of the substrate for further hydrophilization, compared to the electrospinning (the most popular nanofibers production technique). Change of a method for nanofibers spinning results in a shortening of the production of superhydrophilic nanofibrous mats at least two times. PDA coating formation with the addition of sodium periodate provides to the material the ability to absorb water into its structure after a short modification process. This is a much shorter time compared to the process carried out in an alkaline environment without any additional oxidants, other than oxygen from the air, that other authors have previously described for non-fibrous materials modification. We proved that the material superhydrophilic properties persist for at least 28 days—crucial in the design of long-term of the shelf scaffolds for tissue engineering. Furthermore, the modification improves most of the tensile properties of the materials—Young’s modulus and tensile strength—leaving the elasticity unaffected. The applied method of producing nanofibers and their further modification allows for obtaining a superhydrophilic nanofibrous material in less than 3 h, using only a two-step process. Materials obtained in the variant II of the modification processes exhibit superior properties, mentioned above, over that modified using variant I process. What is the most important from the scaffolds design point of view, is that all the investigated PDA-coated PU nanofibrous materials exhibit sped up fibroblast cells adhesion and spreading. Variant I of the modification increases the rate of fibroblast cell adhesion, which is visible in two times faster adaptation of cells to the surface of scaffolds, compared to those modified in variant II (1 h and 2 h of adaptation, respectively).

The presented modification method can be used to hydrophilize various porous materials in a simple, quick, and water-based process. Nanoparticle-based dendritic structures formed on the nanofibers increase the specific surface area of the modified material. The modification process may be the first step to the functionalization of the nanofibrous materials by covalent immobilization of many chemical compounds (e.g., bioactive molecules) to the PDA coating in a simple water-based subsequent reaction. Since most of the fibrous scaffolds are generally hydrophobic, increased wettability and presence of PDA nanostructures offer improved cell seeding efficiency, cell adhesion by additional attachment sites for cells along the fibers, which may be useful, especially in scaffolds for tissue engineering applications.

## Figures and Tables

**Table 1:** Properties of unmodified and modified PU nanofibrous material.

Variant	Modification Time (h)	Mean Fiber Diameter ± SD (nm)	Mean Pore Size ± SD (μm)	Mean Sample Thickness ± SD (μm)	Porosity ± SD (%)
Unmodified PU fibers	-	286 ± 146	3.3 ± 2.3	303 ± 26	83.5 ± 2.4
PU/PDA V-I	2	254 ± 91	3.7 ± 1.9	162 ± 23	61.2 ± 0.9 *
	4	236 ± 81	3.8 ± 2.5	134 ± 16	61.7 ± 3.2 *
	8	256 ± 101	3.4 ± 1.2	181 ± 23	65.6 ± 3.2 *
	24	259 ± 81	3.6 ± 2.0	180 ± 19	65.2 ± 8.7 *
PU/PDA V-II	0.5	259 ± 90	4.5 ± 2.0 *	198 ± 25	70.5 ± 3.9 *
	1	250 ± 83	4.8 ± 2.4 *	209 ± 3	73.5 ± 2.0
	2	289 ± 104	5.4 ± 1.9 *	238 ± 12	72.2 ± 3.8
	4	305 ± 115	5.8 ± 2.1 *	231 ± 6	73.9 ± 1.0

* values statistically different (*p* < 0.01) from the values measured for unmodified nanofibrous materials.

## Supplementary Materials

Supplementary Materials can be found at https://www.mdpi.com/1422-0067/21/18/6798/s1.

## Abbreviations

ASTM	ASTM International, formerly American Society for Testing and Materials
ATCC	American Type Culture Collection
ATR	Attenuated total reflection
CLSM	Confocal laser scanning microscope
DAPI	4′,6-diamidino-2-phenylindole
DMEM	Dulbecco’s modified Eagle’s medium
FBS	Fetal bovine serum
FESEM	Field emission scanning electron microscopy
FTIR	Fourier-transform infrared spectroscopy
hMSCs	Human mesenchymal stem cells
HUVECs	Human umbilical vein endothelial cells
PBS	Phosphate-buffered saline
PCL	Polycaprolactone
PDA	Polydopamine
PDMS	Polydimethylsiloxane
PLGA	Poly(lactic-*co*-glycolic acid)
PLLA	Poly(L-lactic acid)
PU	Polyurethane
SBS	Solution blow spinning
SE(U)	Secondary scattered electrons (the upper detector)
SEM	Scanning electron microscope
V-I	Variant I
V-II	Variant II
XTT	Sodium 3′-[1- (phenylaminocarbonyl)- 3,4- tetrazolium]-bis (4-methoxy6-nitro) benzene sulfonic acid hydrate
