# Supplementary material for: Superhydrophilic Polyurethane/Polydopamine Nanofibrous Materials Enhancing Cell Adhesion for Application in Tissue Engineering
Source: Int J Mol Sci. 2020 Sep 16;21(18):6798. doi: 10.3390/ijms21186798 (PMC7555238; doi:10.3390/ijms21186798)
Supplement: Supplementary file 1 [file ijms-21-06798-s001.zip › Supplementary materials.docx]

Supplementary material for:

**Superhydrophilic polyurethane/polydopamine nanofibrous materials enhancing cell adhesion for application in tissue engineering**

Kamil Kopeć^a^*, Michał Wojasiński^a^, Tomasz Ciach^a,b^

^a^Warsaw University of Technology, Faculty of Chemical and Process Engineering, Biomedical Engineering Laboratory, Waryńskiego 1, 00-645 Warsaw, Poland, [kamil.kopec@pw.edu.pl](mailto:kamil.kopec@pw.edu.pl),

^b^Warsaw University of Technology, Centre for Advanced Materials and Technologies CEZAMAT, Poleczki 19, 02-822, Warsaw, Poland.

Supplementary material for this manuscript includes video files showing recordings of the imbibition effect of water droplets into the nanofibrous polyurethane materials. Video S1 shows a 10x increased in speed imbibition effect on unmodified polyurethane nanofibrous mat. Video S2 and Video S3 show the same effect on PU nanofibrous mats modified using Variant I and Variant II, respectively. The duration of videos corresponds to the measured imbibition time reported in the manuscript. Stills for each video file are presented below, with captions for videos.


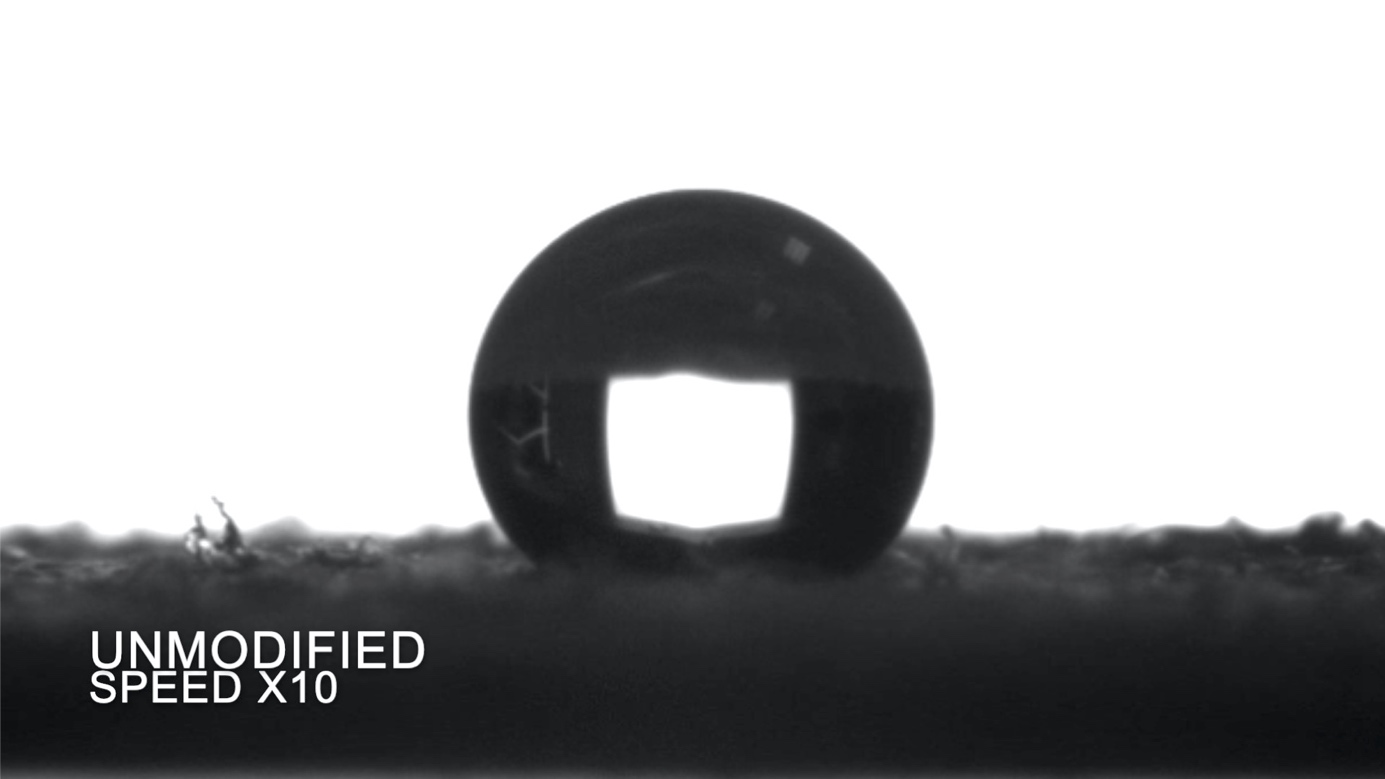


**Video S1.** Imbibition effect of a water droplet on unmodified nanofibrous polyurethane (PU) material. Video is speeded-up 10x.


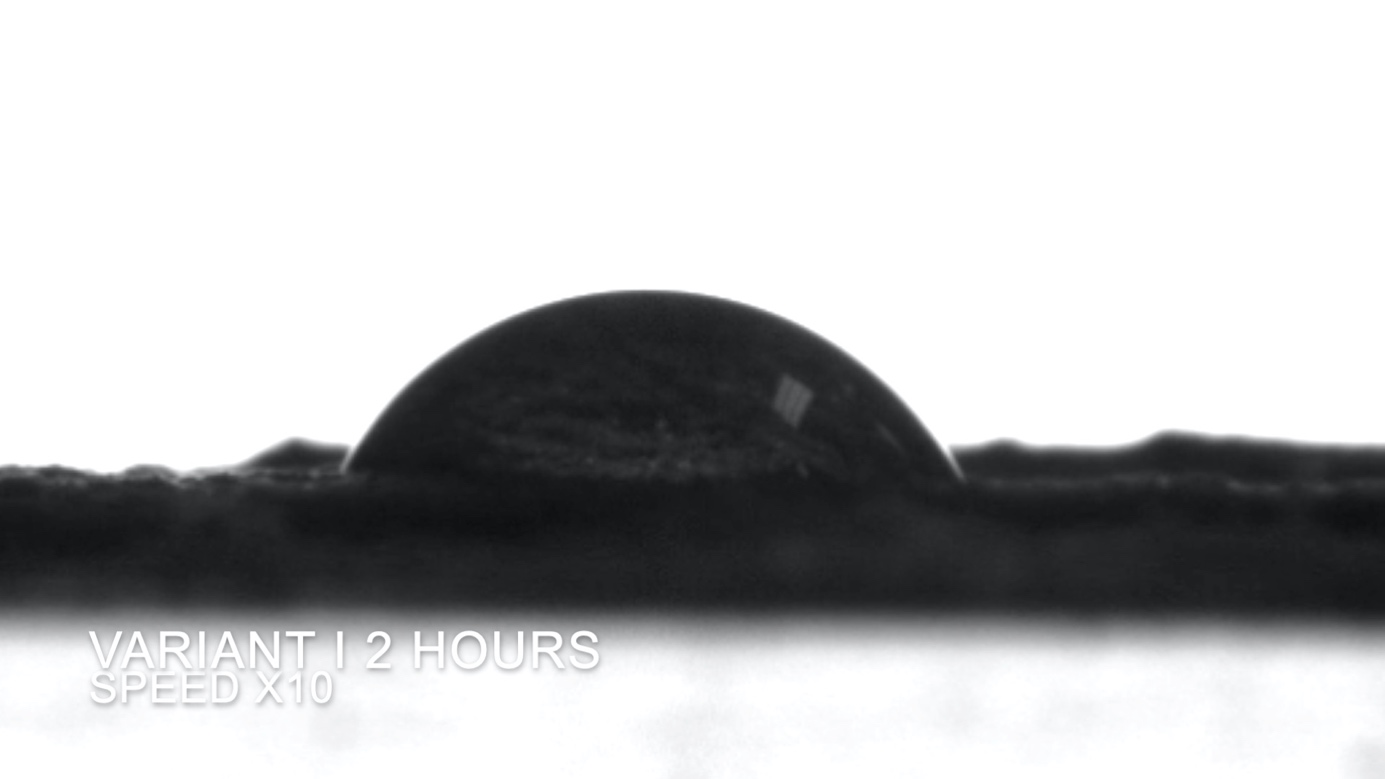


**Video S2.** Imbibition effect of a water droplet on nanofibrous polyurethane (PU) material modified using Variant I (2 hours of modification). Video is speeded-up 10x.


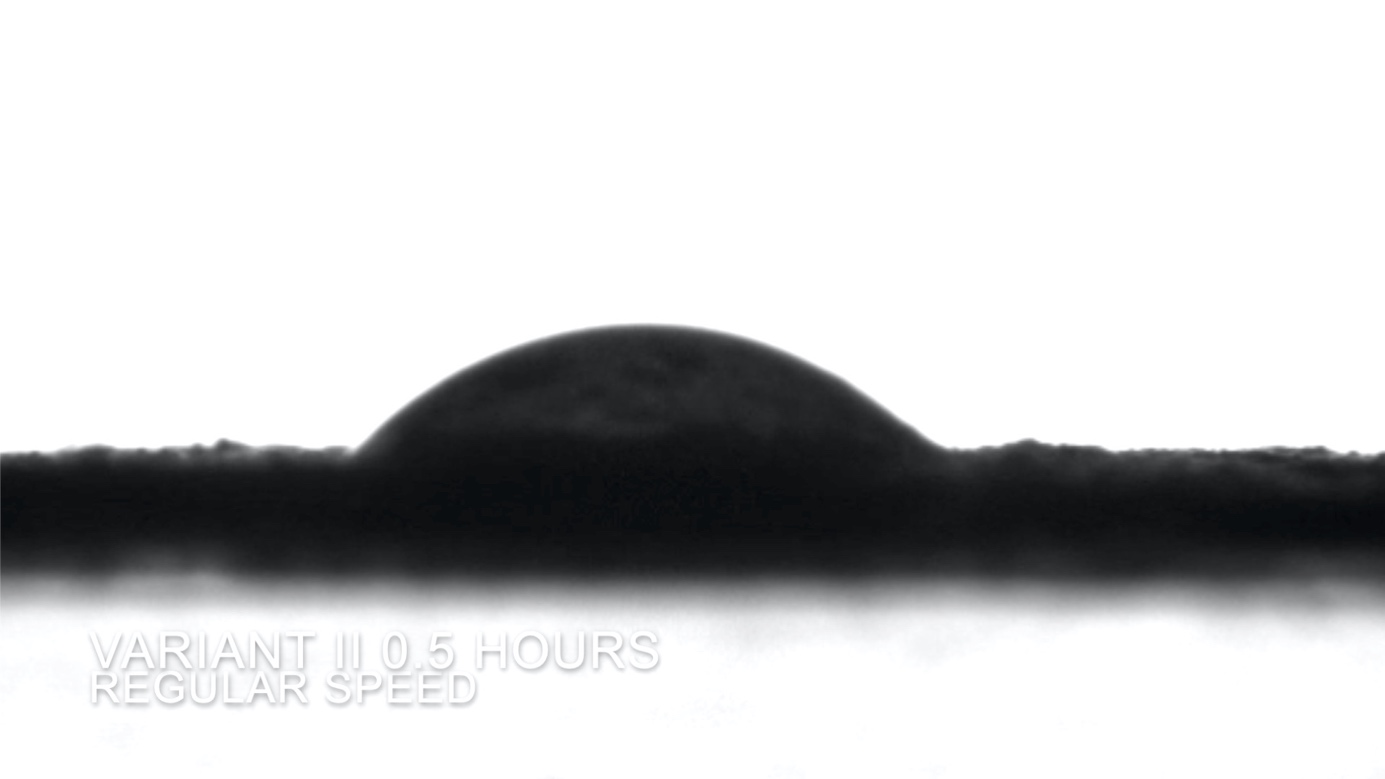


**Video S3.** Imbibition effect of a water droplet on nanofibrous polyurethane (PU) material modified using Variant II (0.5 hours of modification). Video is at the original speed.
